# Network Physiology of Exercise: Beyond Molecular and Omics Perspectives

**DOI:** 10.1186/s40798-022-00512-0

**Published:** 2022-09-23

**Authors:** Natàlia Balagué, Robert Hristovski, Maricarmen Almarcha, Sergi Garcia-Retortillo, Plamen Ch. Ivanov

**Affiliations:** 1 Complex Systems in Sport Research Group, Institut Nacional d’Educació Fisica de Catalunya (INEFC), University of Barcelona (UB), Barcelona, Spain; 2 Complex Systems in Sport Research Group, Faculty of Physical Education, Sport and Health, Ss. Cyril and Methodius University, 1000 Skopje, Republic of Macedonia; 3 Keck Laboratory for Network Physiology, Department of Physics, Boston University, Boston, MA 02215 USA; 4 Department of Health and Exercise Science, Wake Forest University, Winston-Salem, NC 21709 USA; 5 Harvard Medical School and Division of Sleep Medicine, Brigham and Women’s Hospital, Boston, MA 02115 USA; 6 Institute of Biophysics and Biomedical Engineering, Bulgarian Academy of Sciences, 1113 Sofia, Bulgaria

**Keywords:** Network Physiology, Physiological interactions, Horizontal integration, Vertical integration, Multivariate time series analysis, Intra-individual co-variability, Dynamic couplings, Exercise-related processes

## Abstract

Molecular Exercise Physiology and Omics approaches represent an important step toward synthesis and integration, the original essence of Physiology. Despite the significant progress they have introduced in Exercise Physiology (EP), some of their theoretical and methodological assumptions are still limiting the understanding of the complexity of sport-related phenomena. Based on general principles of biological evolution and supported by complex network science, this paper aims to contrast theoretical and methodological aspects of molecular and network-based approaches to EP. After explaining the main EP challenges and why sport-related phenomena cannot be understood if reduced to the molecular level, the paper proposes some methodological research advances related to the type of studied variables and measures, the data acquisition techniques, the type of data analysis and the assumed relations among physiological levels. Inspired by Network Physiology, Network Physiology of Exercise provides a new paradigm and formalism to quantify cross-communication among diverse systems across levels and time scales to improve our understanding of exercise-related phenomena and opens new horizons for exercise testing in health and disease.

## Key Points

- The explanation of macroscopic sport-related phenomena cannot be reduced to molecular levels.
- Physiological states are products of nested dynamics of vertical (among levels) and horizontal (among components of the same level) nonlinear interactions.
- Network Physiology of Exercise provides data analysis techniques to investigate how systems coordinate and synchronize in an integrative way.

## Introduction

The field of Exercise Physiology (EP) studies how the body adapts to acute and chronic exercise stimuli and has an enormous impact on both basic physiology and sports science. Interventions related to training and testing, physical fitness and conditioning, and sports medicine in general are based on empirical and theoretical evidence derived from EP research. Despite the fundamental discoveries, vast progress, and achievements in EP for over a century, the reductionist framework that has traditionally dominated research in the field has imposed limitations to the exploration and understanding of the regulatory mechanisms underlying complex physiological responses to exercise. The investigation of exercise-induced fatigue is a clear example of such limitations. Focusing the attention on molecular processes with few exceptions [1, 2], the explorations in the field have failed to detect a single component or process responsible for exercise-induced fatigue. Thus, it has been hypothesized that multiple processes, systems, and subsystems may play a distributed role in the evolution of fatigue. However, some relevant questions are underexplored: which is the role of cross-communications among systems during the fatigue process, how fatigue manifests at different time scales, how non-proportional events occur during fatigue (e.g., the spontaneous task disengagement), and which are the basic principles of integration and adaptation of the phenomenon? [3–5].

Recent Molecular Exercise Physiology and Omics investigations have extended their scope beyond the study of single tissues or molecular targets to overcome such limitations. Omics focuses on analyzing large pools of biological molecules of certain kinds, such as proteins, metabolites, genes, etc., in a cell, organ, or organism. With technological and computational advances, such approaches seek to map molecular/omics networks of systemic adaptations to exercise in a holistic, unbiased, and integrated manner [6]. Their main objective is to uncover the deeper biological mechanisms underlying human adaptation to exercise, understand better its health benefits, and develop personalized intervention strategies [6–11]. However, the different omics branches such as genomics, transcriptomics, proteomics, metabolomics, etc., refer to the same level of analysis: molecular networks.

There is a tremendous potential for network approaches to better understand the complexity and interconnectedness of molecular processes through genomic and proteomic interactions to (a) assess and quantify biological adaptations to exercise and (b) fill critical gaps in the current understanding of the underlying molecular mechanisms of exercise. However, complex network approaches based on graphs theory are limited to the static representation of genomic and proteomic associations [12, 13]. Molecular Exercise Physiology, in turn, is still dominantly based on the traditional non-dynamic bottom-up group-pooled statistical inference modes of inquiry that have characterized EP research for a century. This research framework does not fully reflect empirical observations that: (i) molecular processes are dynamic and context-dependent, (ii) physiological relations do not operate only bottom-up but also top-down from the entire organism to the molecular level as well as horizontally among systems and processes at a given time scale and level, and (iii) the causal changes may drastically vary among individuals and these differences are not fully detectable from group-pooled statistics.

The challenge of coping with the complexity of sport-related phenomena, emerging from coupling and network interactions among physiological systems and subsystems across spatiotemporal scales, cannot be solved only by focusing on a collective characterization and quantification of pools of biological molecules. Cross-disciplinary collaboration and advancements in both technology and data analytics methods can also be insufficient if reductionist theoretical and methodological research assumptions of EP are kept.

This opinion paper outlines the main challenges in EP and the future directions of research to improve our understanding of the complex mechanisms underlying exercise-related phenomena:

- Find general principles of physiological processes to explain every new context or exercise condition adequately,
- Use general explanatory concepts and principles valid in biology/physiology and other dimensions (e.g., psychology, biomechanics) and levels of matter organization (e.g., social),
- Focus on the physiological processes' dynamics (i.e., changes in time) instead of associations between static measures,
- Investigate multilevel vertical (among molecular, cellular, tissue, organ, etc.) and horizontal interactions among physiological systems to explore multilayer network-based mechanisms underlying the emergence of exercise-related phenomena rather than reducing them to static measures performed at the molecular level.

The reader can find the rationale for these challenges and the directions for future research of EP based in network science in a previous publication [14]. Within the broader framework of the emerging field of Network Physiology [15, 16], Network Physiology of Exercise addresses the fundamental question of how diverse physiological and organ systems across levels continuously coordinate, synchronize and integrate as a network during exercise [17]. This paper will specifically focus on contrasting theoretical and methodological aspects of molecular and network-based approaches to EP.

## Sport-Related Phenomena cannot be Understood if Reduced to a Molecular Level

Life is a property emerging from the interaction of cell components and cannot be explained either by any single component or by their simple aggregation because in biology, “more is different” [18], and relations are as important as entities [19]. Similarly, macroscopic physiological states and functions emerge from the interaction among diverse structures and processes operating at different spatiotemporal scales (from the subcellular level to the entire organism). To understand the mechanisms underlying such integrated states and functions, it is necessary to study the laws of interactions and principles of coordination and integration among physiological systems and subsystems [20, 21] that lead to continuous adaptation to intrinsic and external perturbations [22–27].

New properties that cannot be directly deduced from those of the level below emerge at each physiological level (molecular, cellular, tissue, organ, etc.). Then, macroscopically defined sport-related phenomena (fatigue, injuries, sports performance, etc.) cannot be inferred from simple extrapolation of the properties of elementary molecular components. For instance, one cannot explain exercise-induced fatigue through metabolic substrates or end products [4]. In other words, acute fatigue correlates with biochemical alterations, but such alterations cannot explain the complex disabling symptom limiting performance [1]. Similarly, although decision-making in sports entails electrochemical cell processes, such processes cannot explain how a player decides to shoot or pass a ball.

Understanding the biological behavior at each level requires specific research that is as fundamental in its nature as any other. Paraphrasing Anderson [18], the ability to reduce the organism to microscopic constituents and processes does not imply the possibility of starting from those constituents and reconstructing the organism. This is due to difficulties of scale and complexity. The closer we get to the elementary components, the less relevant they seem to be to the emerging macroscopic phenomena (i.e., manifested at the organism level) of relevance in EP.

**Fig. 1:**
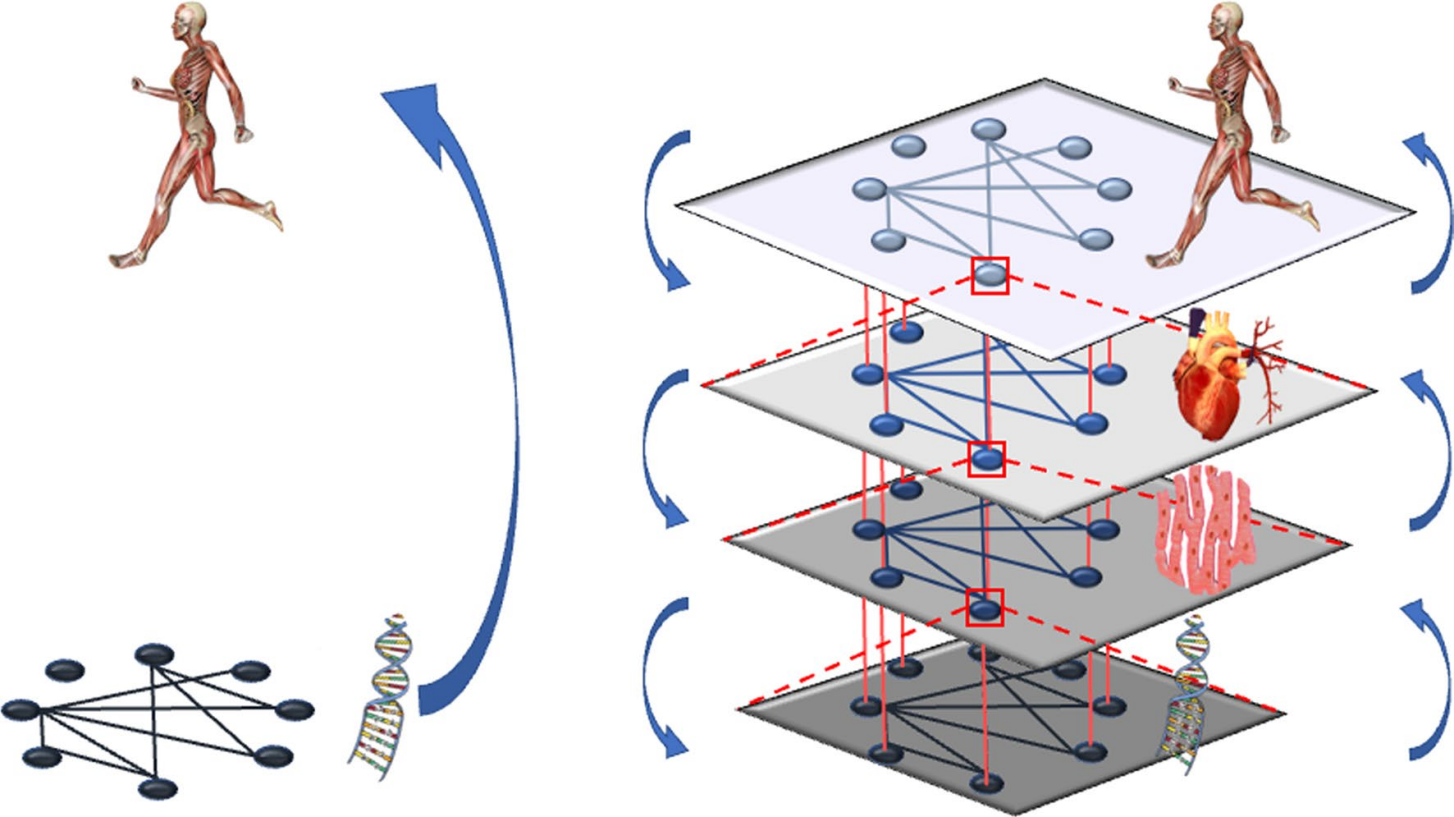
Contrast between Molecular Exercise Physiology (left), focused on non-dynamical bottom-up statistical inference techniques, and Network Physiology of Exercise (right), focused on the nested dynamics of the vertical and horizontal physiological network interactions

Hence, future research in EP has to address several fundamental questions like (a) how hierarchical integration among physiological systems is produced across levels, that is, how to re-compose the previously decomposed (through reductionism) physiological mechanisms, (b) how the entire ensembles capture key emergent properties, and (c) how to provide an integrated and synthetic explanation of sport-related phenomena [28]. Physiological processes operating at different levels, which are hierarchically self-organized, interact dynamically through circular causality (see Fig. 1 right, vertical axis) [29]. Bottom-up, cell properties emerge from molecules and organelles interactions, tissue properties from cells interactions, and organs and systems (cardiovascular, pulmonary, hormonal, etc.) properties from tissue interactions. Top-down, and due to circular causality, such organs and systems constrain the behavior of the levels below [30]. For example, tissue stress factors (tissue level) constrain intercellular communication and individual cell mechanics and signaling (cellular level), which further constrain gene expression (molecular level) [31]. That is, physiological interactions function horizontally (among components of each level) and vertically (among different levels) and in two directions, not only bottom-up [20]. In turn, it is the whole organism, not just the cellular and molecular levels, which interacts with the environment (e.g., adapting to the exercise workload requirements).

The nature of this organizational hierarchy of physiological processes has not been a research subject in sports science, and its description is usually simply anatomic. It is worth noting that Fig. 1 (right) represents vertically the organizational levels and the causal interactions across levels. Certainly, it is not a strict requirement that one level can interact only with its own closest neighbor (both vertically and horizontally). For instance, molecules do not interact only within their original cells; through the bloodstream they may interact with molecules of other organs. However, those interactions are still at the molecular level.

Although correlated, the operating timescale of each physiological level is different. For instance, cellular metabolic adaptations occur faster (e.g., enzymes need a few weeks) than adaptations at the organ level (e.g., heart volumes need several weeks) and organismic level (e.g., cardiovascular fitness needs a few months). Although it is often assumed that molecular and cellular changes are the most relevant in physiology, social and psychological constraints, changing more slowly in time, have a more permanent effect [32]. For instance, acute fatigue is permanently constrained by the socially induced habits of athletes (e.g., nutrition, sleep, working hours, etc.).

Instead of focusing on a microscopic molecular level, Bizzarri et al. [19] propose a mesoscopic approach, increasingly popular in the epistemology of biology [33, 34], for conceptualizing, investigating and explaining co-emergence in physiology. Mesoscopic variables properly capture some physiological function and simultaneously constrain the behavior of microscopic level variables, yet allowing a wide variety of configurations (see the above example of tissue stress factors). Operating at a sufficiently higher level of organization than the microscopic processes, mesoscopic variables are largely insensitive to the microscopic level fluctuations. In other words, the same value of the mesoscopic variable can be attained by an ever-changing variety of microlevel component configurations (see below the explanation of concepts of biological degeneracy and pleiotropy). Bizzari et al. 2019 (p.3) [19] provide the following analogy: “A useful architectural analog of the mesoscopic level are the arches of a gothic cathedral: the arch occupies the intermediate layer between the stone and the entire building and represents the optimal level where to study the forces responsible for the stability of the cathedral as a whole”. The arch network of push–pull momenta is the relevant level to explain a cathedral's stability. Moreover, the vectors of mesoscopic forces constrain the mechanical stress of bricks and their substructures. Hence, to understand the stress state of bricks (microlevel) and the stability state of the cathedral (macrolevel), one has to consult the structure and dynamics of arch networks (mesoscopic level). Thus, explanations and predictive possibilities may be maximal at the mesoscopic level of physiological function rather than at the microscopic level of organization. For instance, the cardiac rhythm, a system property arising from the coordinated activity of the cardiac muscle, does not pertain to any single myocardial cell or single molecule, but may inform about a change of physiological state induced by exercise (e.g., athlete’s bradycardia). This mesoscopic way of thinking is also popular in ecology [35] and in biological network-based approaches [36], for example, in networks with time-evolving interactions [37].

The Dynamic Systems Theory (DST) conceptual framework (e.g., stability, instability, phase transition) [22, 38–42] can be adopted to explain the dynamic functional integration of the physiological networks during exercise, including its multilevel patterns. The components and the subsystems may lose their properties when the whole system is destabilized through exercise. For instance, as a consequence of accumulated effort, a fatigue-induced spontaneous task disengagement occurs [5, 43]. Thus, to better understand sport-related phenomena, it is recommended to assume a multilevel network physiology perspective [20, 21], which considers the hierarchical organization of structures and processes information flows through the levels instead of focusing only on the molecular level.

The progressive integration of the DST framework within Molecular and Cell Biology, Genomics and all Omics approaches [13, 44–48] at the forefront of science in recent decades represents an important step forward in the direction of synthesis and integration in Physiology. However, the relational ontology of biological systems goes beyond graph representation of static genomic and proteomic associations at the microscopic level [19, 20].

Complex systems science, born with integrative scientific purposes, and searching common principles among substance organization levels, provides concepts able to re-compose the decomposed through reductionism, and physiological mechanisms and integrate the fragmented information [49]. Recognized properties of complex adaptive systems (CAS) like synergies, self-organization, circular causality, nonlinear dynamic interactions and criticality, scale invariance and universality [50], generally ignored in EP, should be introduced to improve the understanding of exercise-related phenomena.

## Biological Intelligence and General Principles of Complex Adaptive Systems as a Theoretical Approach in EP

In CAS, the adaptation properties are based on biological intelligence, defined as the capacity to evade or escape states of reduced functional solutions [51]. Many biological mechanisms attain this capacity by generating flexible behaviors at the edge of instability [26, 41, 50–55]. Such solutions involve multiple levels and scales: from the subcellular (biophysical, biochemical) to the integrated organism level (physiological subsystems, systems, organs) and global sociological behaviors (groups of individuals).

In EP, the emergence of adaptation properties has been described as a tendency to prevent states of reduced fitness, that is, states of reduced functional diversity potential [14, 51]. This refers to the richness of functional synergies [56, 57] and fast recovery time after a perturbation. Intelligent behavior may be expressed at diverse levels and timescales. Hristovski and Balagué [51] offer the following example: At short timescales, the bouts of intense exercise produce acute fatigue that decreases the diversity potential of the organism. At a long-term scale, however, systems across all levels—from the molecular to the entire organism—react by temporarily increasing their diversity potential in anticipation of possible incoming perturbations. These biological behaviors have been modeled as strong anticipation phenomena [58, 59].

These exercise effects can mainly compensate for the tendency of aging and disease to reduce the diversity potential of individual physiological systems and the entire organism [60, 61]. The growth of biological intelligence in the context of CAS requires diverse time-varying and adaptive coupling forms among physiological systems [16, 22, 23, 25, 62], as well as adaptive responses to challenging and stimulating environments to evade temporary stagnation, which may, on a longer time scale, turn into decreasing functional diversity potential and spontaneous collapse [51]. This underlines the enormous potential of exercise for developing functional diversity.

The function of biological intelligence is to allow physiological systems and organisms to escape states with reduced diversity potential, such as aging, fatigue, injuries or other unexpected perturbations. This is done by creating new synergies among systems, which may include new dimensions, not only those related to exercise modalities [51]. Exercise is not the only intervention that may increase the functional diversity potential and/or evade states of reduced fitness [63]. For instance, internal regulatory mechanisms (e.g., sleep) play an essential role in restorative functions at systems (e.g., cardiac, respiratory, locomotor) and organism levels through increased physiologic variability and temporal organization across scales [36, 42, 64].

Biological intelligence is linked to properties of CAS, which are commonly not considered in the theoretical framework of Molecular Exercise Physiology research as:

*Spontaneous formation of synergies:* flexible patterns of coordination arising from local or global interactions between parts [65], which form emergent structures and functions that compensate each other to satisfy the physiological demands of exercise. They act at all levels governing, in turn, the systems’ behavior [33, 66]. While computer scientists build programs that tell circuits what to do, nature creates synergies [67, 68]. In EP, it is common to propose conceptual models where the primary regulators and programmers are the Central Nervous System (CNS) (see, e.g., [9, 69]) or the DNA. However, CAS does not need any internal or external programmer to regulate its functions [50, 70]. Properties of such functions (i.e., stability, instability, variability, switches among states, criticality etc.) are parametrically regulated in both the CNS and the DNA [71]. This means that physiological conditions emerge from the interaction among multilevel system components (the CNS being another component) through a self-organized process. The search for the ultimate high-level regulator would end in infinite regress (what regulates the regulator of the regulator, etc.), and such an approach cannot lead to understanding the basic principles of network integration and control of underlying exercise-related physiological phenomena.

Self-organized dynamics may play a crucial role in phenotypic traits. Molenaar [72] discusses a third possible source of phenotypic variation besides the two well-known ones: the genotype and the environment [48]. The developmental dynamics itself may be the third source. The initial differences in twin embryos may be enhanced during pre and postnatal development to bring about significant phenotypical differences in monozygotic twins [73].

*Pleiotropy:* The same components and processes may be assembled to produce multiple functions. For instance, the skeletal muscle, with genuine/primordial contractile functions, may also exert immunological and endocrine functions [74, 75].

*Degeneracy:* Different components produce the same function, and different synergies may be activated to attain the same task goal [76, 77]. For instance, motor units cooperate and reciprocally compensate for their activation over several timescales to perform a functional motor action over time during a running competition. Pleiotropy and degeneracy enable CAS to switch between diverse coordinative states [78].

*Nonlinear dynamics:* The interactions among parts are such that non-proportional effects to perturbations may arise [50, 79]. For instance, a slight change in neurotransmitters may produce a significant change in the pattern of neuronal activity [80].

Readers can contrast further theoretical research postulates of the current EP paradigm from the framework of Networks Science [14, 81, 82].

## Contrasting Methodological Approaches of Molecular Exercise Physiology and Omics with Network Physiology of Exercise

Molecular Exercise Physiology and Omics approaches focus on data collection to catalogue exercise-regulated pathways [6, 7, 11]. As shown in Fig. 1 (left), these approaches study interactions at the molecular level and establish non-dynamical bottom-up group-pooled statistical inferences about the entire person (e.g., performance level or health status). In contrast, Network Physiology of Exercise integrates bottom-up and top-down (circular causality) vertical and horizontal network levels (Fig. 1, right). It avoids the gap between micro and macro structures and functions, considering synergies between components of the same level and synergies between levels of the physiological network.

Network Physiology of Exercise emerged as a new area of research inspired by the multi-disciplinary field of Network Physiology [15, 16, 20–22, 83, 84] to transform EP's theoretical and methodological assumptions, the research program, and the practical issues derived from the evidence-based research. Network Physiology addresses how physiological systems and subsystems coordinate, synchronize, and integrate their dynamics to optimize functions at the organism level and maintain health. It aims at uncovering the biological mechanisms regulating the dynamics of individual systems and their network interactions [15, 23–25, 84]. It satisfies both (i) the mechanistic requirement of structure and localization (e.g., nodes and edges/links in dynamic networks representing localized integrated organ systems, subsystems, components or processes, and interactions among them across various levels in the human organism), and (ii) the requirement of dynamical invariance and generality that is enabled by dynamical systems approach [28, 85].

Identifying and quantifying functional forms of coupling, studying network interactions among diverse physiological systems and processes, and finding the mechanisms of causality and network control are significant challenges due to the complex dynamics of organ systems [64, 86–89]. Such complexity arises from intrinsic interactions of multi-component cellular and neuronal subsystems that build and regulate each organ in the human body, leading to intermittent, scale-invariant, and nonlinear output signals. This is further compounded by various coupling and feedback interactions between organ systems that continuously vary in time [90], the nature of which is not understood. It was recently discovered that two organ systems could communicate through several forms of coupling that simultaneously coexist [22, 91]. This poses a challenge to understand how organs integrate their functions to generate emergent behavior of the human body as a single entity able to adapt to internal and external perturbations and maintain homeostasis [92].

In contrast to traditional complex network theory, where edges/links are constant and represent static graphs of association, novel approaches in Network Physiology take into consideration (i) the complex dynamics of individual systems (network nodes), (ii) dynamical aspects of network links representing organ communications in real time, (iii) the evolution of organ interactions with time, and (iv) the emergence of collective network behavior in response to changes in physiologic states and conditions.

Table 1 contrasts the current methodological traits of Molecular Exercise Physiology and Omics with those that characterize Network Physiology of Exercise.

**Table 1:** Contrasts between methodological traits of molecular exercise physiology and omics with those that characterize network physiology of exercise

Methodological traits	Molecular Exercise Physiology and Omics	Network Physiology of Exercise
Variables	Molecular mechanisms and networks	Networked meso- or macroscopic collective variables
Data acquisition	Group-pooled data	Intra-individual multiple time series
Measures	Means and max values of variables	Connectivity/Transfer entropy/Mutual information/Phase coherence/Coupling functions/Phase synchronization/Time-delay stability
Analysis	Population to individual generalization	Individual to population generalization
Relations	Bottom-up (from micro to macro levels) static group-pooled statistical inferences	Bottom-up and top-down (circular causality) multilevel dynamic interactions

The methodological steps followed by Molecular Exercise Physiology and Omics approaches are similar to those followed by EP. For instance, in correlational studies: (a) measuring N physiological variables on K number of subjects, (b) calculating the group-pooled Pearson correlations matrices, and (c) performing some form of data reduction technique. This group-pooled data analysis ignores the critical caveat that results obtained at the group level cannot be unconditionally interpreted as relations or effects that exist at the individual level [72].

In contrast, genuine network analysis research follows the following steps: (a) analysis of a set of time series at the individual level and formulation of individual networks, (b) check for general features of networks among individuals, and (c) determine the degree of generalization of certain network features at a population level. It also detects and discusses individual-specific characteristics.

Instead of using only molecular data to establish bottom-up statistical timeless inferences from micro to macroscopic phenomena (e.g., relating molecular maps to fatigue states or performance), Network Physiology of Exercise proposes the study of the time-variability properties of mesoscopic or macroscopic behavioral variables extracted at action level (e.g., see [5]). The study of the dynamics of such variables may inform during exercise about the vicinity of qualitative non-proportional changes in the system like transitions from stable to unstable states, critical behavior (critical slowing down, flickering, enhancement of fluctuations), and phase transitions/bifurcations (as occur in the fatigue-induced spontaneous task disengagement) [37, 43]. It is worth mentioning that physiological states do not correspond to fixed quantitative values of parameters or set points but can emerge from diverse constellations of quantitative values [3, 5, 81, 93]. Several interaction-based measures like connectivity, entropy, phase coherence, Hurst exponent, Lyapunov exponent, etc. can be used to detect such behavior [15, 23, 25, 62, 94, 95–101].

Another crucial methodological difference refers to the analysis of the acquired physiological data. Most of the research on Molecular Exercise Physiology and Omics approaches infer intra-individual phenomena from the study of inter-individual variations obtained through group-pooled data. This approach has some fundamental methodological issues that should be discussed in detail. The main aim of EP research, performed at any level of analysis, is to find the regulatory mechanisms that occur at the intra-individual (i.e., organism) level as an effect of exercise. The intra-organismic processes, not the population, are the explanatory target. It is important to note here that intra-individual variability and co-variability unfold in time and must be measured through time series analytical tools.

While the problem of sample to population generalization has been much discussed, investigated, and used in inferential statistics, much less attention has been focused on the justification of sample or population to individual generalization. A tacit assumption has been that the results obtained at the sample and generalized to the population level are representative of the changes of a ‘typical’ [i.e., average) individual [72, 102]. In other words, the group-pooled data would merely enhance the typical phenomenon in each individual.

Pooling-over group subjects is the predominant research practice in EP, particularly in exercise and health-related research. Even the state-of-the-art software packages for time series analysis [103] are based on pooling-over-subjects approaches. However, the generalization of results from population to individual (or between clusters of individuals) is not necessarily valid for CAS [72]. The causal changes may drastically vary from individual to individual. For instance, the network structure of certain methylation properties obtained at the group-pooled level may not exist at the individual level, but be just a group-pooled statistical artifact. In other words, the group-pooled, inter-individual co-variability may substantially differ from the real intra-individual co-variability [104, 105]. These fundamental research problems require a complete change in traditional experimental designs and a transition to multivariate time series analysis of individuals. This change will open up the path to bridging the nomothetic and idiographic approaches in EP [106] and will help in the detection of the conditions in which group-pooled effects may meet the true intra-individual effects. In this sense, the traditional Molecular Exercise Physiology and Network Physiology of Exercise can be seen as complementary research approaches.

## Conclusions

In recent years, research in EP has made significant strides toward Molecular Exercise Physiology and the implementation of novel Omics technologies to investigate exercise-related phenomena. While molecular and Omics approaches to EP have led to substantial new insights, exploring the effects of exercise at the macroscopic organism level cannot be reduced to the molecular levels only. Group-pooled data analysis, characteristic of EP research, can produce statistical population artifacts of cause–effect relations that may not exist at the individual level. Thus, such group-pooled results cannot be directly generalized to the macroscopic organism level without knowing the causal processes and network interactions at the intra-individual mesoscopic scales.

In addition to focusing on microlevel molecular and Omics processes, recent developments in Network Physiology have utilized novel empirical and modeling approaches based on adaptive networks of dynamical systems to open new horizons. These involve exploration of coupling forms and network dynamics of physiologic interactions and investigation of the mechanisms of integration at the meso- and macroscopic levels, where the emergence of order is of physiological relevance for exercise-related phenomena.

Such dynamic network approaches to EP at the meso- and macroscopic levels are essential to (i) uncover laws of cross-communication and causality among systems, (ii) establish principles of adaptation and hierarchical integration, and (iii) build a new mechanistic picture of the control processes underlying the emergence of exercise-related physiological states and functions, and (iv) facilitate the classification and prediction of behaviors at the organism level in response to exercise, based on dynamic maps of network interactions among physiological and organ systems.

The future challenges of EP to go beyond molecular and Omics approaches are the following: (i) focus on general principles of physiological processes to understand adequately every new exercise context, (ii) use general concepts and principles derived from DST and networks of dynamical systems, valid for all levels of substance organization, to conceptualize, investigate and explain exercise-related phenomena, (iii) focus on nonlinear dynamic interactions instead of static associations, and (iv) provide multilayer network-based phenomenological explanations of exercise-related phenomena as products of nested dynamics of nonlinear interactions within vertical (among levels) and horizontal (among components at the same level) integration.

Network Physiology of Exercise, a new branch of Network Physiology, has emerged to explain physiological states and functions as products of such nested network dynamics. Novel data analysis techniques have been developed and successfully utilized to investigate how physiological processes and systems coordinate and synchronize as a network. Such approaches can lead to a new class of evaluation tools to assess the effects of exercise. Specifically, building the theoretical formalism for Network Physiology of Exercise will facilitate the development of network-based biomarkers, able to track how physiological states evolve under exercise settings and improve our knowledge about the mechanisms underlying diverse exercise-related phenomena.

## Data Availability

Not applicable.
